# Functional MRI Readouts From BOLD and Diffusion Measurements Differentially Respond to Optogenetic Activation and Tissue Heating

**DOI:** 10.3389/fnins.2019.01104

**Published:** 2019-10-24

**Authors:** Franziska Albers, Lydia Wachsmuth, Daniel Schache, Henriette Lambers, Cornelius Faber

**Affiliations:** Translational Research Imaging Center, Department of Clinical Radiology, University Hospital Münster, Münster, Germany

**Keywords:** functional diffusion, optogenetics, BOLD, heating artifacts, small animal MRI

## Abstract

Functional blood-oxygenation-level-dependent (BOLD) MRI provides a brain-wide readout that depends on the hemodynamic response to neuronal activity. Diffusion fMRI has been proposed as an alternative to BOLD fMRI and has been postulated to directly rely on neuronal activity. These complementary functional readouts are versatile tools to be combined with optogenetic stimulation to investigate networks of the brain. The cell-specificity and temporal precision of optogenetic manipulations promise to enable further investigation of the origin of fMRI signals. The signal characteristics of the diffusion fMRI readout vice versa may better resolve network effects of optogenetic stimulation. However, the light application needed for optogenetic stimulation is accompanied by heat deposition within the tissue. As both diffusion and BOLD are sensitive to temperature changes, light application can lead to apparent activations confounding the interpretation of fMRI data. The degree of tissue heating, the appearance of apparent activation in different fMRI sequences and the origin of these phenomena are not well understood. Here, we disentangled apparent activations in BOLD and diffusion measurements in rats from physiological activation upon sensory or optogenetic stimulation. Both, BOLD and diffusion fMRI revealed similar signal shapes upon sensory stimulation that differed clearly from those upon heating. Apparent activations induced by high-intensity light application were dominated by T_2_^∗^-effects and resulted in mainly negative signal changes. We estimated that even low-intensity light application used for optogenetic stimulation reduces the BOLD response close to the fiber by up to 0.4%. The diffusion fMRI signal contained T_2_, T_2_^∗^ and diffusion components. The apparent diffusion coefficient, which reflects the isolated diffusion component, showed negative changes upon both optogenetic and electric forepaw stimulation. In contrast, positive changes were detected upon high-intensity light application and thus ruled out heating as a major contributor to the diffusion fMRI signal.

## Introduction

Functional MRI (fMRI) has become the most important neuroimaging modality, since it offers non-invasive, brain-wide imaging of neuronal activity with high spatial and temporal resolution. A number of different MRI sequences offer complementary readouts of physiological parameters, which are used as surrogate for activation of defined regions of the brain (Hoge et al., 1999; Lu et al., 2003; Liu and Brown, 2007; Lu and van Zijl, 2012). Signal changes in blood-oxygenation-level-dependent (BOLD) fMRI (Kwong et al., 1992; Kim and Ogawa, 2012); Buxton, 2013) rely on the hemodynamic response and therefore represent an indirect readout depending on the neurovascular coupling between neuronal activity and vascular response. As potential alternative readout, diffusion fMRI, which measures alterations of water diffusivity in the brain, has been claimed to directly reflect neuronal activation (Le Bihan et al., 2006; Le Bihan, 2007). The mechanisms behind diffusivity changes are not well understood. One theory proposes that neuronal cells swell during activation, thereby hindering diffusion in the tissue, and, consequently, creating a signal change in diffusion-weighted fMRI sequences (Le Bihan, 2007; Tsurugizawa et al., 2013; Abe et al., 2017a). Faster onset and decay times for the diffusion-weighted signal compared to BOLD were reported and attributed to a more direct neuronal correlation. Data supporting cell swelling have been obtained for Alpysia Californica neurons (Abe et al., 2017b). However, controversies exist regarding the existence and the origin of diffusion fMRI signal changes (Miller et al., 2007; Jin and Kim, 2008; Autio et al., 2011; Bai et al., 2016). Despite this dissent about functional diffusion MRI, this readout appears suitable to investigate the neurophysiological response to optogenetic stimulation of neuronal circuitry of the brain.

The implementation of optogenetic tools has enabled precise activation or inhibition of genetically defined cell populations, which has revolutionized the field of neuroscience. Experiments with unprecedented specificity, inhibiting or activating defined components of sensory networks have become possible (Boyden et al., 2005; Fenno et al., 2011; Kim et al., 2017; Repina et al., 2017). For comprehensive understanding of the effects of optogenetic manipulation of networks of the brain, a brain-wide readout modality is required. Task-based BOLD fMRI has previously been combined with optogenetic stimulation (Lee et al., 2010; Desai et al., 2011; Anenberg et al., 2015; Liang et al., 2015; Takata et al., 2015; Albers et al., 2018b). While most aspects of sensory stimulation have been found to be reproduced by optogenetic stimulation (Iordanova et al., 2015; Schmid et al., 2016), some studies have observed slight differences between sensory and optogenetic stimulation (Uhlirova et al., 2016; Albers et al., 2018a). Functional diffusion MRI as potentially more direct neuronal readout of optogenetic stimulation, therefore, appears promising. Vice versa, optogenetic control of neuronal activity may offer novel insights into the mechanism behind signal changes in functional diffusion MRI.

One major concern when using optogenetic tools is that the application of light inherently comprises the risk of local heat deposition in tissue, which may give rise to unwanted effects. Already low light intensities cause blood flow changes under certain anesthetics (Rungta et al., 2017). Low light intensities applied continuously even suppress neuronal activity of medium spiny neurons and inhibitory interneurons, and potentially have direct impact on motor behavior (Owen et al., 2019). Higher light intensities may additionally lead to optical stimulation of the eyes and retina (Schmid et al., 2017). When using MRI as readout modality, the situation is further aggravated. The MRI signal itself is highly temperature dependent and even slight temperature changes due to light application result in heating artifacts in functional images, depending on light intensity, light color and MRI sequence (Christie et al., 2013; Schmid et al., 2017).

In this study, we have combined optogenetic stimulation with a diffusion fMRI readout and aimed to separate signal changes due to heating artifacts from those due to the neurophysiological response. We have assessed and compared signal changes in BOLD and diffusion fMRI upon sensory stimulation, optogenetic stimulation, and tissue heating. In the first set of experiments we characterized heating artifacts both *in vivo* and *ex vivo*. Then, we compared functional signal time courses upon tissue heating with those upon sensory and optogenetic stimulation *in vivo*. Finally, we characterized the functional diffusion signal comparing time courses from BOLD- and diffusion-type measurements upon optogenetic stimulation.

## Materials and Methods

All experiments were carried out according to the German Tierschutzgesetz and were approved by the Landesamt für Natur, Umwelt und Verbraucherschutz of Nordrhein-Westfalen, Germany (84-02.04.2015.A427). Experiments were performed in 21 Fisher rats (F344) *in vivo*. 20 of these were female, one male rat was used in experiment 1 (light application). Animals were 10–27 weeks old at the time of the experiment and weighed between 152 and 201 g (females, the one male weighted 288 g). Additionally, measurements were performed in 3 Fisher rats *ex vivo*, two of which had also been measured *in vivo*. Rats were housed in groups of 2-3 animals under a regular light/dark schedule (12/12 h). Food (Altromin 1324) and water were available *ad libitum*.

### Animal Preparation

All animal preparations and surgical procedures were performed under isoflurane anesthesia (5% for induction, 2-3% for preparations). For stable expression of the opsin C1V1_TT_ in optogenetic experiments, 5 rats were injected with the viral vector rAAV2/CamKIIa-C1V1-(E122T/E112T)-TS-eYFP [gift from Karl Deisseroth, Addgene plasmid # 35499; http://n2t.net/addgene:35499; RRID:Addgene_35499 (Yizhar et al., 2011)] at least 4 weeks prior to experiments. Injection site relative to bregma was AP + 0.2 mm, LR – 2.4 mm, DV 1.6 mm, 35° lateral from midline in the sensory cortex forelimb area. Directly before the experiment a 200 μm optic fiber was implanted above the opsin expressing region (AP + 0.2 mm, LR – 3.3 mm, DV 0.3 mm) and attached to the skull with UV glue as described previously (Schmid et al., 2016).

Animals were ventilated (MRI-1 Ventilator, CWE Inc., Ardmore, PA, United States) and received a muscle relaxant (Pancuronium 2 mg/kg bolus followed by continuous injection of 1.5 mg/kg^∗^h; or Atracurium 5 mg/kg bolus followed by continuous injection of 5 mg/kg^∗^h) during MR imaging. Expiratory CO_2_ was monitored (CapStar-100 CO_2_ Analyzer, CWE Inc., Ardmore, PA, United States) and kept at 2.4 ± 0.4%. Body temperature was also monitored and kept at 37 ± 0.7°C.

For functional imaging anesthesia was switched from isoflurane to medetomidine (s.c. bolus injection of 0.04 mg/kg followed by a continuous infusion of 0.05 mg/kg^∗^h). After the medetomidine bolus isoflurane was discontinued within 10–15 min. 40 min after the medetomidine bolus functional imaging started.

### fMRI

fMRI was performed in all rats using BOLD fMRI and diffusion fMRI. All scans were acquired on a 9.4 T small animal MRI (Biospec 94/20, Bruker BioSpin GmbH, Ettlingen, Germany) using a linearly polarized resonator and a surface coil with 2 cm (experiment 1) or 1 cm (experiments 2, 3) diameter. In the first experiment we wanted to examine the full possible extent of the heating artifacts, accordingly the large 2 cm coil, which almost covered the complete brain, was used. In the following experiments the smaller coil was used to gain maximum signal to noise ratio for measurements with high temporal resolution. First, a T_2_-weighted anatomical image was acquired in the same position as the following functional scans (RARE sequence, TR/TE_eff_ 2000/50 ms, RARE factor 8, 256 × 256 matrix, 110 × 100 μm^2^ resolution, 1.2 mm slice thickness, 9 contiguous slices) to verify the fiber position.

#### Experiment 1: Identifying the Threshold for Heating Artifacts

First, a threshold for heating artifacts was determined to have safe light intensity limits for optogenetic stimulation (see also Schmid et al., 2017) and to use sufficient light intensities for high-intensity light application to evoke heating artifacts. GE-BOLD was most sensitive to heating artifacts, accordingly, results from GE-BOLD were used to determine a safe limit for optogenetic stimulation. Experiments were performed in 5 naïve animals with GE-EPI with TR 1000 ms, TE 18 ms, FA 60°, 350 × 325 μm^2^ resolution, 1.2 mm slice thickness, 9 slices, EPI readout time 29.44 ms. These data were analyzed using a *U*-test and additionally using an analysis without significance testing as described in sections “*U*-Test Analysis” and “Additional Analysis Without Significance Testing for Experiment 1.”

#### Experiment 2: High-Intensity Light Application

High-intensity light application was performed in 11 naïve rats *in vivo*. Light application was also applied in 3 naïve animals *ex vivo*, two of which had previously been measured *in vivo*. For *ex vivo* scans rats were transcardially perfused with PBS and ice-cold formalin (3.7%) solution after the *in vivo* experiment. *Ex vivo* rats were either measured directly or stored frozen at −18° and measured (unfrozen) at a later time. Green laser light (552 nm) was applied via a 200 μm optic fiber with a paradigm of 5 s stimulation (100 ms-pulses, 9 Hz, 217 mW/mm^2^ for *in vivo* and 217–354 mW/mm^2^ for *ex vivo* experiments) and 25 s rest. This resulted in mean light intensities (maximum light intensity at fiber tip × duty cycle) of 195–319 mW/mm^2^.

In this study the term high-intensity light application always refers to light application with mean light intensity of 195 mW/mm^2^ or higher in naïve animals.

BOLD fMRI was performed using GE-EPI (TR 250 ms or 1000 ms, TE 18 ms or 35 ms, FA 30° or 60°, 350 × 325 μm^2^ resolution, 1.2 mm slice thickness, 3 or 9 slices, EPI readout time 29.44 ms) and SE-EPI (TR 250 ms or 1000 ms, TE 35/35.85 ms, FA 90°, 350 × 325 μm^2^ resolution, 1.2 mm slice thickness, 3 or 9 slices, EPI readout time 29.44 ms) sequences. Diffusion fMRI was performed using single SE diffusion-weighted EPI sequences (TR 250 ms or 1000 ms, TE 35.85 ms, *b* = 500/1000/1500 s/mm^2^, Δ = 15 ms, δ = 2 ms, FA 150° or 125°, 350 × 325 μm^2^ resolution, 1.2 mm slice thickness, 2-3 or 9 slices, EPI readout time 25.6 ms). The EPI readout times in the diffusion sequences were in the range of T_2_^∗^ (25 ms) in the rat brain. Accordingly, these sequences were T_2_^∗^-weighted, as well as diffusion-weighted. For diffusion and GE-BOLD sequences the flip angle was adjusted to obtain maximum signal. The data from experiment 2 were analyzed using a *U*-test and additionally using a general linear model approach as described in sections “*U*-Test Analysis” and “GLM Analysis.”

Throughout the manuscript the sequences are referred to as GE-BOLD, SE-BOLD and diffusion fMRI. The term GE-BOLD always refers to the sequence with TE 18 ms unless the long TE is explicitly stated.

#### Experiment 3: Optogenetic and Electric Forepaw Stimulation

Optogenetic stimulation was performed in 5 rats expressing the opsin C1V1_TT_ in excitatory neurons of the sensory cortex forelimb area. Electric stimulation was performed in 8 naïve and opsin-expressing rats. Optogenetic stimulation with green laser light (552 nm) was performed using a paradigm of 5 s stimulation (10 ms-pulses, 9 Hz, 162–217 mW/mm^2^ at fiber tip) and 25 s rest. This resulted in a mean light intensity (maximum light intensity at fiber tip x duty cycle) of 14.6–19.5 mW/mm^2^. For electric stimulation electrodes were inserted into the forepaw and 1 ms-pulses with 1–1.5 mA at 9 Hz were applied using the same paradigm. fMRI measurements were performed as in experiment 2. The data from experiment 3 were analyzed using a *U*-test and additionally using a general linear model approach as described in sections “*U*-Test Analysis” and “GLM Analysis.”

### Analysis

Data were analyzed in two ways. In both cases activated voxels were determined with a statistical test and time courses were extracted from those. The first approach was model-free using a *U*-test to determine activated voxels. The second approach relied on the commonly applied general linear model (GLM) using the software package SPM 12 (Statistical Parametric Mapping, Functional Imaging Laboratory, Wellcome Trust Centre for Neuroimaging, London, United Kingdom).

#### *U*-Test Analysis

fMRI data were preprocessed (realigned and resliced) using SPM 12. Data were realigned in a two-step procedure. Data were not registered to a template, instead each set of images was realigned separately to its own reference. First, the images were realigned to the first image of the time series for each slice and a mean image of each slice was calculated. In a second step images were aligned with that mean. In some measurements with 3 slices the reslice operation cut the edge slices away. In those cases, unprocessed data were used for further analysis. For this data slight variations between consecutive images may reduce the observed BOLD response, however, the unsuccessful alignment reduced the response even more. After realignment, the data were analyzed with a custom-written script in MATLAB (Release 2017b, The MathWorks, Inc., Natick, MA, United States).

Raw fMRI data were subjected to a *t*-test using ImageJ (Schneider et al., 2012) comparing rest and stimulation periods (with a 2 s delay to account for the delayed hemodynamic response) to generate activation maps. Significantly (*p* < 0.001, no Bonferroni correction) activated voxels were color coded and overlaid on the mean EPI images.

In each slice that showed activation in the *t*-test a region of interest (ROI) covering the activation and adjacent pixels was drawn. In each voxel of this ROI the temporal signal to noise ratio (tSNR) was calculated by dividing the mean signal during rest periods by the standard deviation of the signal during rest periods. tSNR values were averaged across the ROI to obtain a mean voxelwise tSNR for each measurement.

To find significantly activated (positive or negative) voxels, a voxelwise *U*-test was performed in the ROI. The *U*-test was performed between rest and stimulation periods, which were shifted by a 2 s delay to account for the delay of the hemodynamic response. Significance was assumed at *p* = 0.05 after Bonferroni correction was applied. Result of the *U*-test was a binary activation map with 1 coding for an activated voxel and 0 for a non-activated voxel.

When at least 5 voxels were active, data from activated voxels were averaged across voxels, across stimulation trials and across animals, separately for voxels with positive and negative signal changes. The limit of 5 voxels was chosen arbitrarily before the beginning of the analysis. Resulting averaged time courses were normalized to the pre-stimulus baseline. For all time courses mean + SEM (standard error of the mean, $S\,E\,M=s\,t\,a\,n\,d\,a\,r\,d\,d\,e\,v\,i\,a\,t\,i\,o\,n/\sqrt{\#\,o\,f\,s\,a\,m\,p\,l\,e\,s}$) is shown.

In the standard analysis the stimulation period was shifted by 2 s to account for the slow BOLD response. This increased the detection sensitivity when the signal change subsided slowly after the end of the stimulus (Supplementary Figure 1). To confirm that the shifted analysis did not change signal characteristics such as time to peak, we performed an additional *U*-test without any shift. The latter procedure resulted in less activated voxels. We did not see any significant changes in time to peak behavior between shifted and non-shifted analysis.

Normalized time courses were calculated by dividing signal by the maximum signal in the respective time course. Area-weighted time courses were calculated by multiplying the single time courses from each animal with the number of activated voxels before averaging. Signal onsets in the averaged time courses were defined as first measured value above 15% of the maximum signal. Time to peak and time to baseline were calculated on the averaged time courses. Time to peak was defined as time from the onset of stimulation to the peak. Time to baseline was determined as time from the onset of stimulation until the baseline was reached again after the peak. The baseline was defined as values closer to zero than 0.05% for positive changes or −0.05% for negative changes.

The apparent diffusion coefficient (ADC) was calculated from measurements with different *b*-values. For b1 > b2 the ADC is given by *ADC* = *ln*(*S_b2_/S_b1_*)/(*b1−b2*). *S_b1_* is the signal from measurements with b1. The error was calculated according to the propagation of uncertainty to be:$\Delta \,A\,D\,C=\frac{-\Delta \,S_{b\,1}}{S_{b\,1}\,\left(b_{1}-b_{2}\right)}+\frac{\Delta \,S_{b\,2}}{S_{b\,2}\,\left(b_{1}-b_{2}\right)}$, with Δ*S*_*b*1/2_ the SEM of *S*_*b1/2*_. For the ADC calculation all measurements belonging to one condition e.g., electric stimulation with *b* = 500 s/mm^2^ were summed up. Then, ADC values were calculated for electric stimulation, optogenetic stimulation and light application. For each stimulation the *b*-value combination with the largest difference was chosen, i.e., *b* = 500 s/mm^2^ with *b* = 1000 s/mm^2^ for electric stimulation and, *b* = 500 s/mm^2^ with *b* = 1500 s/mm^2^ for optogenetic stimulation and light application. All shown ADC changes are smoothed by a moving average filter that averaged four measurement points together.

Averaged net activation maps were calculated with MagnAn (BioCom GbR, Uttenreuth, Germany) to compare activation patterns upon high-intensity light application. First, all EPI measurements were registered to one EPI that was chosen as reference. For registration, data were translated and rotated but not warped or scaled. The calculated affine matrices were then applied to the binary activation maps generated with the MATLAB *U*-test analysis. This was done separately for the positive and negative activation maps. During this transformation binary values were allowed to be smeared across voxels to allow a closer registration. Then, the averaged (absolute) negative changes were subtracted from the averaged positive signal changes to obtain net signal changes. Lastly, this net signal change map was overlaid over the reference EPI.

*U*-test analysis was applied to experiments 1, 2 and 3.

#### GLM Analysis

Classically, regression analysis of fMRI data models the expected BOLD response as the convolution of the stimulation paradigm and the canonical hemodynamic response function (HRF). Data upon electric and optogenetic stimulation were analyzed with the canonical HRF. As the canonical HRF implemented in SPM is modeled on human data and evolves much slower than our observed responses, we used an HRF modeled on rodent fMRI data (Lambers et al., 2019). However, as the responses upon light stimulation did not look like the typical BOLD response these data were analyzed with the finite impulse response model (FIR) that offers more freedom for the shape of the response.

Data were realigned and resliced as described in section “*U*-Test Analysis” and additionally smoothed with a 0.5 mm Gaussian kernel. For experiment 2 with high-intensity light application the finite impulse response (FIR) analysis in SPM was performed. A window length of 10 s and an order of 10 was chosen. Data were analyzed for positive and negative responses separately. For experiment 3 with electric and optogenetic stimulation the HRF analysis in SPM was performed. In both cases the family-wise error was used to correct for multiple comparison.

The resulting thresholded maps showed the activated voxels and were used to extract the time courses from these voxels. Again, time courses were only calculated when at least five active voxels were found in the measurement. Subsequently, time courses were treated as described above. Averaged and area-weighted time courses and ADC changes were calculated. Mean activation maps were only calculated based on the *U*-test analysis.

In the main manuscript Figures 2–5, 8, 9 show results of the GLM analysis.

#### Additional Analysis Without Significance Testing for Experiment 1

Data from experiment 1 were also used to estimate the effect of low-intensity light application during optogenetic stimulation on the BOLD response. Even when signal changes do not reach statistical significance, they may diminish the resulting BOLD signal. To extract the largest effect on the BOLD response, a small ROI (circa 1 × 2 mm) covering the area directly around the fiber was chosen. In this ROI data were averaged across all voxels and across stimulation trials to obtain time courses for single measurements. All measurements from one light application condition (i.e., 19.5 mW/mm^2^, 29 mW/mm^2^ etc.) were averaged to obtain mean time courses for different light intensities. For each averaged time course the peak amplitude was extracted. The amplitudes were plotted against the used light intensities and fitted with a linear fit that was forced to include the origin (0, 0).

## Results

In this study we performed functional MRI using GE-BOLD, SE-BOLD and diffusion sequences to compare heat-induced apparent brain activation with sensory or optogenetic stimulation-induced physiological brain activation. Secondly, the functional diffusion signal was characterized using different challenges: high-intensity light application to generate heating artifacts, electric paw and optogenetic stimulation.

### Threshold for Heating Artifacts

**FIGURE 1:**
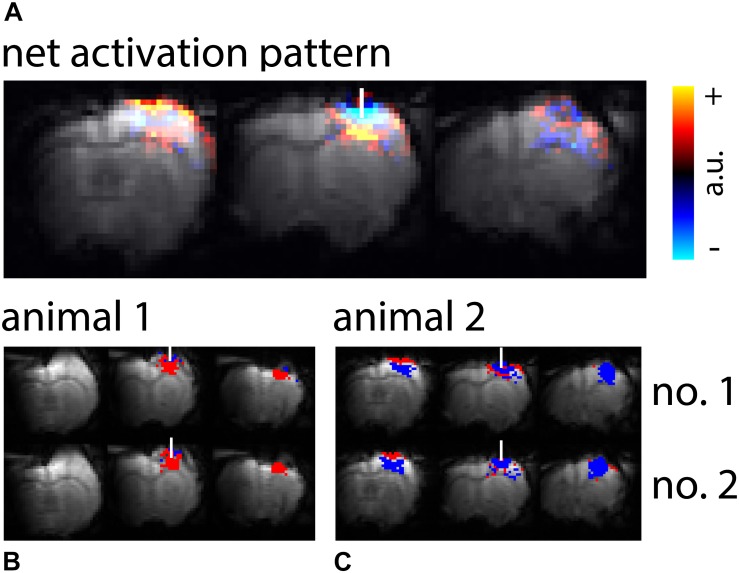
Activation patterns upon high-intensity light application. **(A)** Averaged net signal changes from GE-BOLD upon high-intensity light application were overlaid on the reference EPI. 18 measurements (TR 250 ms) from 7 animals were averaged. **(B,C)** Exemplary maps from two animals, with two ten minutes measurements each (no. 1 and no. 2), are shown. EPIs were overlaid with binary activation maps calculated from the *U*-test with 2 s shift. Red colored voxels indicate areas of significant positive signal change, blue colored voxels indicate areas of significant negative signal change. The white bar indicates the fiber position.

In the first set of experiments safe limits for light intensities applied during optogenetic stimulation, which did not cause heating artifacts, were determined. Heating artifacts were identified by negative signal changes or at higher light intensities by negative and positive signal changes in areas close to the fiber (Figure 1A). With mean light intensities up to 19.5 mW/mm^2^ no heating artifacts were observed with our 2 s shift *U*-test analysis. Light intensities of 156 mW/mm^2^ were sufficient to cause heating artifacts in all GE-BOLD, and 195 mW/mm^2^ in SE-BOLD and diffusion measurements (Table 1).

**TABLE 1:** Signal changes in GE-BOLD upon light stimulation with increasing light intensity in 5 naïve rats.

**Mean light intensity/mW/mm^2^**	**Pixel with negative signal change observed**	**Pixel with positive signal change observed**
19.5	No signal change in *n* = 4	No signal change in *n* = 4
29	−1.6% in *n* = 1/5	No signal change in *n* = 5
39	−2.1% in *n* = 4/5	No signal change in *n* = 5
78	−2.2% in *n* = 5/5	1% in *n* = 1/5
117	−3.4% in *n* = 5/5	1.2% in *n* = 3/5
156	−3.5% in *n* = 5/5	1.2% in *n* = 5/5
195	−4.7% in *n* = 4/4	1.4% in *n* = 4/4

**n* gives the number of measurements with activation/all measurements.*

### Diffusion fMRI With High Temporal Resolution Is Feasible

To enable better characterization of the signal time courses, we implemented functional MRI acquisitions with TR of 250 ms. In diffusion and GE/SE-BOLD fMRI measurements the mean voxelwise tSNR decreased with decreasing TR. For GE-BOLD tSNR decreased from 76 ± 12 at TR 1 s (optogenetic stim., *n* = 6) to 44 ± 5 at TR 250 ms (*n* = 7). For diffusion fMRI with *b* = 1000 s/mm^2^ tSNR decreased from 25 ± 3 at TR 1 s (optogenetic stim., *n* = 4) to 7.3 ± 0.3 at TR 250 ms (*n* = 5). However, even at TR 250 ms the tSNR was sufficient to detect signal changes upon high-intensity light application, optogenetic stimulation and, less robustly, upon electric forepaw stimulation (mean activation maps are shown in Supplementary Figures 3, 5). Of note, these values represent lower limits for tSNR, since calculation was performed across all voxels in the ROI, which sometimes included areas with signal voids.

Tables 2, 3 summarize number and ratio of functional measurements that detected activation for *U*-test analysis and GLM analysis, respectively. In general, detection sensitivity was highest for GE-BOLD, lower for SE-BOLD and diffusion with *b*-value of 500 s/mm^2^, and lowest for diffusion sequences with high *b*-values. Interestingly, detection sensitivity was particularly low for electric stimulation using diffusion sequences. Concerning heating artifacts, negative signal changes were more pronounced in all fMRI sequences and positive signal changes almost absent in diffusion fMRI with high *b*-values.

**TABLE 2:** Results of *U*-test analyses.

	**GE**	**GE long TE**	**SE**	**Diff *b* = 500 s/mm^2^**	**Diff *b* = 1000 s/mm^2^**	**Diff *b* = 1500 s/mm^2^**
Electric	19/19 (17/19)	na	na	12/21 (10/21)	3/21 (1/21)	na
Optogenetic	7/7	na	6/7	6/7	5/7	4/5 (3/5)
High-int. light (neg., *in vivo*)	18/18 (17/18)	19/19	13/19	14/17	14/17 (11/17)	14/22 (11/22)
High-int. light (pos., *in vivo*)	18/18	19/19	7/19 (12/19)	2/17 (6/17)	1/17 (0/17)	0/17 0/17
High-int. light (neg., *ex vivo*)	8/8	5/5	4/5 (2/5)	7/7 (6/7)	7/7 (6/7)	4/4
High-int. light (pos., *ex vivo*)	7/8	5/5	1/5	4/7	4/7 (3/7)	2/4

*The ratio of successful measurements with TR 250 ms for all experimental conditions is shown. Measurements in which more than 5 significantly activated voxels were detected were considered successful. Results of the 0 s shift *U*-test are given in brackets, if different from the 2 s shift result. Conditions that were not experimentally realized are marked with not applicable (na).*

**TABLE 3:** Results of GLM analyses.

	**GE**	**GE long TE**	**SE**	**Diff *b* = 500 s/mm^2^**	**Diff *b* = 1000 s/mm^2^**	**Diff *b* = 1500 s/mm^2^**
Electric	19/19	na	na	15/21	12/21	na
Optogenetic	7/7	na	7/7	7/7	6/7	4/5
High-int. light (neg., *in vivo*)	17/18	19/19	14/19	16/17	16/17	18/22
High-int. light (pos., *in vivo*)	18/18	19/19	10/19	10/17	4/17	2/17
High-int. light (neg., *ex vivo*)	8/8	5/5	5/5	7/7	7/7	4/4
High-int. light (pos., *ex vivo*)	7/8	5/5	1/5	4/7	3/7	2/4

*The ratio of successful measurements with TR 250 ms for all experimental conditions is shown. Measurements in which more than 5 significantly activated voxels were detected were considered successful. Experiments with electric or optogenetic stimulation were analyzed with the HRF, experiments with light application with the FIR. Conditions that were not experimentally realized are marked with not applicable (na).*

For optogenetic and electric stimulation, as well as for negative signal changes upon high-intensity light application, our 2 s shift *U*-test analysis was always equal or more sensitive than the 0 s shift *U*-test analysis, as expected from the shape of the response (Supplementary Figure 1). Only for two conditions with positive signal changes upon high-intensity light application the *U*-test without shift detected more activated voxels (Table 2). The GLM analysis always revealed more activated voxels than the *U*-test analysis, resulting in more measurements meeting the 5 voxel criteria (Table 3). Averaged amplitude was lower for GLM-based time courses, likely because more weakly activated voxels were included. However, as more active voxel were found, the area-weighted amplitude was higher for GLM-based time courses (Supplementary Figure 2 and Supplementary Table 1).

### Spatial Pattern and Temporal Shape of Signal Changes Upon High-Intensity Light Application

High-intensity light application resulted in both voxels with positive and voxels with negative signal changes. Signal changes were most pronounced in GE-BOLD (Tables 2, 3 and Supplementary Figure 3). Group averaged net signal changes resulted in a pattern showing negative signal changes in the vicinity of the fiber tip and positive signal changes more remote from the fiber tip (Figure 1A). While activation patterns in GE-BOLD images differed substantially between animals, repeated measurements produced similar spatial patterns in each animal (Figures 1B,C).

**FIGURE 2:**
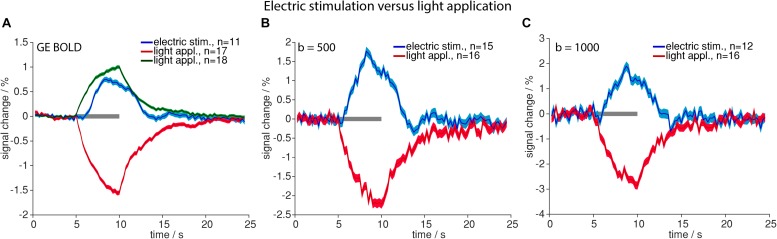
Comparison of high-intensity light application and electric stimulation. Averaged time courses upon electric stimulation (blue) and light application (positive signal changes in green and negative signal changes in red) for GE-BOLD **(A)**, diffusion with *b* = 500 s/mm^2^ **(B)** and diffusion with *b* = 1000 s/mm^2^ **(C)** are shown. Gray bars indicate stimulation periods, mean ± SEM is shown, n indicates number of measurements. All mean time courses are based on time courses extracted with the GLM analysis.

Time courses of signal changes were markedly different for high-intensity light application as compared to electric stimulation. Upon light application signal started to increase immediately, following an exponential saturation curve, and instantly started to decrease exponentially after end of light application. Electric stimulation by contrast resulted in a delayed signal increase according to the well-known HRF (Figure 2).

**FIGURE 3:**
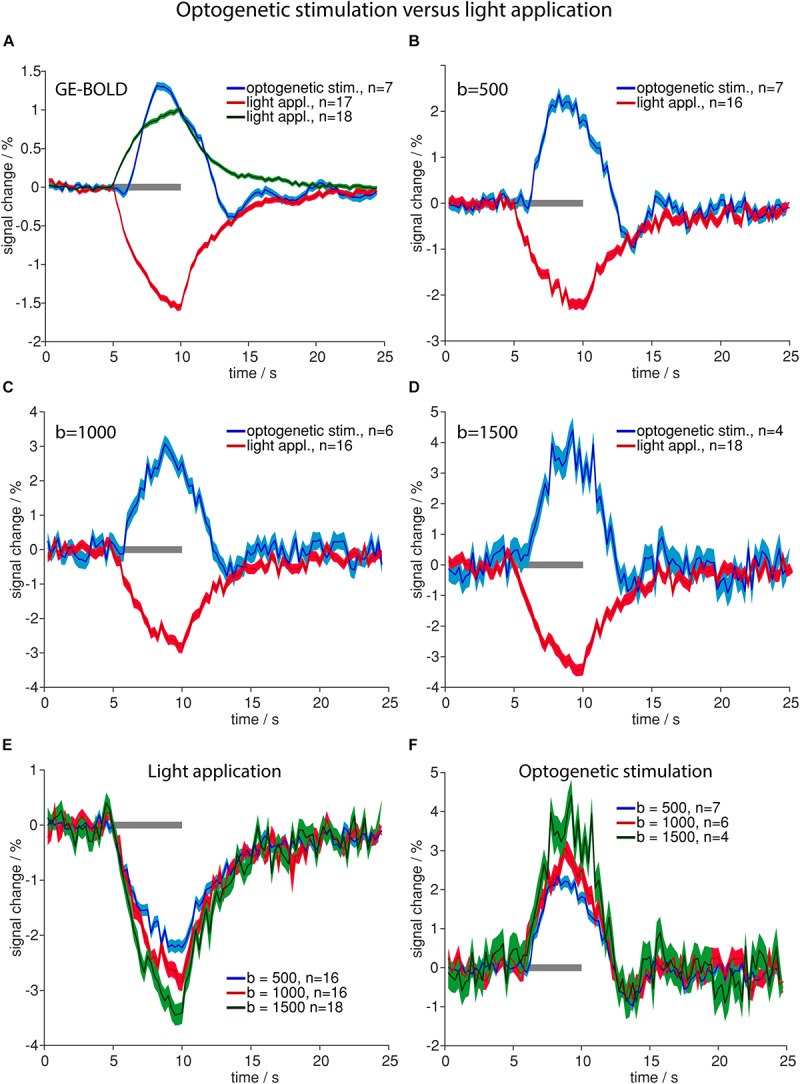
Comparison of high-intensity light application and optogenetic stimulation. **(A–D)** Averaged time courses upon optogenetic stimulation (blue line in **A–D**) and light application (positive changes in green in **A**, negative changes in red in **A–D**) for GE-BOLD **(A)**, diffusion with *b* = 500 s/mm^2^ **(B)**, diffusion with *b* = 1000 s/mm^2^ **(C)** and diffusion with *b* = 1500 s/mm^2^ **(D)** are shown. Signal change upon light application begins with the stimulation and signal rises until stimulation stops. In contrast, signal change upon optogenetic stimulation begins delayed and reaches its peak before the end of stimulation. **(E)** Averaged signal changes upon high-intensity light application for diffusion sequences with *b* = 500 s/mm^2^ (blue), 1000 s/mm^2^ (red) and 1500 s/mm^2^ (green) are shown. **(F)** Averaged signal changes upon optogenetic stimulation for diffusion sequences with *b* = 500 s/mm^2^ (blue), 1000 s/mm^2^ (red) and 1500 s/mm^2^ (green) are shown. In both, **(E,F)**, amplitude rises with increasing diffusion weighting. All time courses were acquired with TR 250 ms. Gray bars indicate stimulation periods, mean ± SEM is shown, n indicates number of measurements. All mean time courses are based on time courses extracted with the GLM analysis. High-intensity light application was always performed in naïve animals that did not express opsins.

Optogenetic stimulation revealed the same signal time course shapes compared to electric stimulation (Figure 2) in GE-BOLD and diffusion measurements (Figures 3A–D). Also in diffusion measurements optogenetic stimulation resulted in a typical hemodynamic response-like signal change, while tissue heating resulted in immediate signal increase and decrease at beginning and end of the illumination, respectively. Times to peak were earlier for responses upon electric or optogenetic stimulation compared to light application (Table 4 and Supplementary Table 2). A *t*-test revealed the significance of these differences (GE electric versus GE light, *p* < 0.0001; *b* = 500 electric versus *b* = 500 light, *p* = 0.0001; and *b* = 1000 electric versus *b* = 1000 light, *p* = 0.01).

**TABLE 4:** Time to peak and time to baseline values for mean time courses based on GLM-analysis are shown.

	**Time to peak**	**Time to baseline**
	**s**	**s**
GE el	3.50 ± 0.16	12.75 ± 0.60
*b* = 500 el	3.25 ± 0.21	13.00 ± 0.90
*b* = 1000 el	3.75 ± 0.36	12.50 ± 1.20
GE opto	3.25 ± 0.12	12.50 ± 0.48
SE opto	3.25 ± 0.18	12.50 ± 0.70
*b* = 500 opto	3.25 ± 0.21	12.50 ± 0.80
*b* = 1000 opto	3.75 ± 0.30	12.25 ± 1.00
GE light	4.75 ± 0.14	21.50 ± 0.62
GE long TE light	4.50 ± 0.09	24.25 ± 0.50
SE light	5.00 ± 0.32	15.50 ± 1.00
*b* = 500 light	5.00 ± 0.26	21.75 ± 1.12
*b* = 1000 light	5.00 ± 0.28	20.50 ± 1.14
*b* = 1500 light	4.50 ± 0.25	15.50 ± 0.86

*Error of time to peak and time to baseline was estimated by the relative error determined by the SEM of the peak amplitude.*

Averaged amplitudes of signal changes were larger with diffusion sequences than with GE-BOLD sequences (Figures 3A–D). Amplitudes also increased with larger *b*-values for both optogenetic stimulation and high-intensity light application (Figures 3E,F).

Amplitudes of negative signal changes upon high-intensity light application were larger than amplitudes of positive signal changes for all sequences (Supplementary Table 1). In diffusion sequences negative signal changes in heating artifacts increased with increasing *b*-value and were −2.2 ± 0.1% for *b* = 500 s/mm^2^, −2.9% ± 0.2 for *b* = 1000 s/mm^2^, and −3.5 ± 0.2% for *b* = 1500 s/mm^2^ (Figure 3E).

While averaged amplitudes upon optogenetic stimulation and high-intensity light application were higher in diffusion sequences (Figures 3A–D), the number of detected voxels showing signal changes was higher in GE-BOLD compared to diffusion measurements. To assess this effect area-weighted time courses were calculated by multiplying the single time courses from each animal with the number of activated voxels before averaging (Supplementary Figure 4). Area-weighted signal changes were markedly larger for GE-BOLD than for diffusion sequences.

### Similar Signal Changes Due to Tissue Heating Occur *in vivo* and *ex vivo*

**FIGURE 4:**
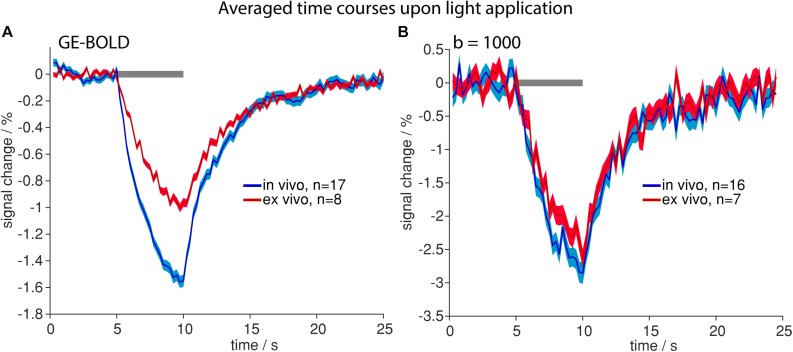
Time courses upon light application *in vivo* (blue) and *ex vivo* (red). Averaged time courses upon high-intensity light application in GE-BOLD **(A)** and diffusion fMRI with *b* = 1000 s/mm^2^ **(B)** are shown. Time courses were acquired with TR 250 ms. Gray bars indicate stimulation periods, mean ± SEM is shown, n indicates number of measurements. All mean time courses are based on time courses extracted with the FIR GLM analysis.

To assess the impact of blood flow on the detected signal change during tissue heating, we performed the same experiments *ex vivo*. Similar spatial patterns and temporal shapes of signal changes were observed in dead rats with both GE-BOLD and diffusion weighted measurements (Figure 4 and Supplementary Figure 3). While for GE-BOLD the amplitude of signal changes was 1.56 times larger *in vivo*, nearly identical time course and amplitude were observed for the diffusion measurement with *b* = 1000 s/mm^2^ (−2.64% *ex vivo* and −2.86% *in vivo*), suggesting that the contribution of blood flow is less pronounced in diffusion fMRI.

### Signal Changes Upon Tissue Heating Are Dominated by T_2_^∗^-Effects

Since markedly smaller areas of signal change upon light application were observed in diffusion sequences (which have a SE preparation) as compared to GE-BOLD (Supplementary Figures 2, 3, 4), we assessed the impact of sequence differences in more detail. Both *in vivo* and *ex vivo*, we compared GE-BOLD with standard TE of 18 ms, GE-BOLD with long TE of 35 ms and SE-BOLD. Negative signal changes were more pronounced in all fMRI sequences. Positive signal changes were almost absent in diffusion fMRI with high *b*-values based on the *U*-test analysis (Table 2) and less prominent based on the GLM analysis (Table 3). Accordingly, the analysis was solely focused on negative signal changes.

**FIGURE 5:**
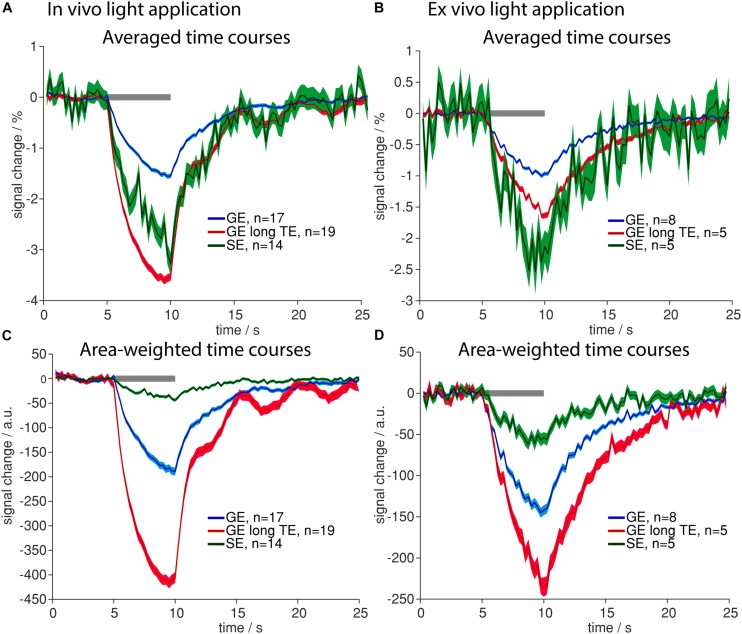
Sensitivity to heating artifacts in different fMRI sequences. **(A,B)** Averaged time courses upon light application *in vivo* **(A)** and *ex vivo* **(B)** for GE-BOLD with 18 ms TE (blue), GE-BOLD with 35 ms TE (red) and SE-BOLD (green) are shown. **(C,D)** Area-weighted time courses upon light application were calculated *in vivo* **(C)** and *ex vivo* **(D)** for GE-BOLD with short TE (blue), GE-BOLD with long TE (red) and SE-BOLD (green). Gray bars indicate stimulation periods, mean ± SEM is shown, n indicates number of measurements. All mean time courses are based on time courses extracted with the FIR GLM analysis.

Largest averaged amplitudes of signal changes were observed in GE with long TE in *in vivo* measurements and in SE measurements *ex vivo*, smallest amplitudes were observed for GE-BOLD with short TE (Figures 5A,B). For GE-BOLD with long TE, amplitudes were similar to SE *in vivo* (−3.61 and −3.26%) and factor 1.53 smaller than SE *ex vivo* (Figures 5A,B), where blood had been removed prior to the measurement.

**FIGURE 6:**
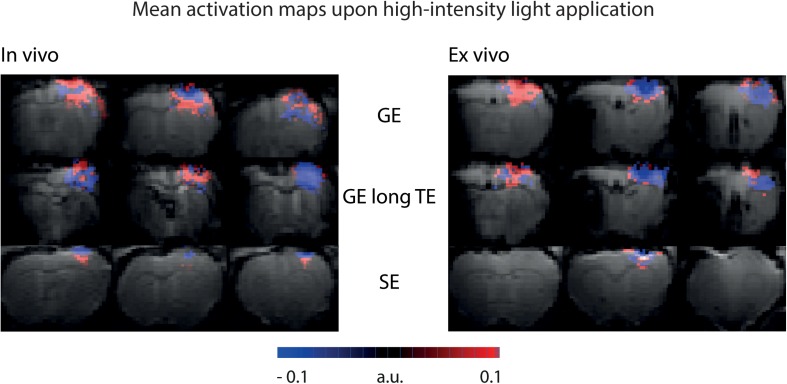
Mean activation maps upon high-intensity light application. Mean activation maps from GE sequence, GE sequence with long TE and SE sequence are shown for *in vivo* and *ex vivo* experiments. Mean activation maps are based on activation maps from the *U*-test analysis with 2 s shift. Maps are color-coded in a range that makes all activated voxels visible.

To be sensitive for both amplitude of signal changes and the number of significant voxels, area-weighted time courses were calculated by multiplying the single time courses with the number of activated voxels before averaging. With GE-BOLD with TE 35 ms we found the largest areas of signal change, and consequently, the largest area-weighted changes, while with SE-BOLD the smallest areas of negative signal change and area-weighted changes were found both *in vivo* and *ex vivo* (Figures 5C,D). Mean activation maps also showed larger areas of positive and negative signal change for GE sequences compared to the SE sequence (Figure 6) and diffusion sequences (Supplementary Figure 3). These data show that, although T_2_ changes may lead to pronounced local signal changes, T_2_^∗^ is the dominating mechanism for the observed changes in most of the voxels.

### Light Application Can Reduce the BOLD Signal Already at Low Light Intensities

**FIGURE 7:**
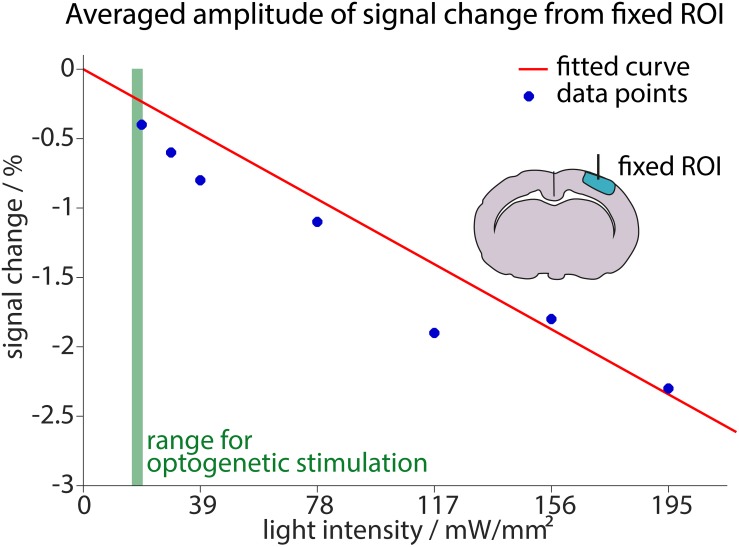
Estimation of signal reduction due to light application. The averaged maximum amplitudes of signal changes in a small ROI around the fiber are plotted against the applied light intensity (blue dots). The linear fit including these measurements and the origin (0, 0) is shown in red. Fit result was *f*(x) = –0.013x (95%-confidence intervals between –0.01509 and –0.01068 for the fit). Measurements were performed for light intensities ranging from 19.5 mW/mm^2^ to 195 mW/mm^2^. For reference, the range of light intensities used for optogenetic stimulation in this study (14.6–19.5 mW/mm^2^) is shown in green.

Next, we assessed whether heating induced signal changes may interfere with physiological BOLD signal. For this purpose, we revisited our initial control experiments, which had identified light intensities up to 19.5 mW/mm^2^ as safe limit not inducing detectable heating artifacts. However, the initial analysis employed significance testing, which introduced a threshold, classifying part of the weak heat-induced signal changes as not significant. Yet, such sub-threshold signal changes, not detected as heating artifacts, may occur, even at the low light intensities as used for optogenetic stimulation and may diminish the BOLD response. In order to estimate this possible effect, the initial control measurements were reanalyzed without significance testing, by defining a small ROI directly around the fiber, where the strongest effects of the light were expected. Data were averaged across the ROI and across stimulation trials. Amplitudes of negative signal changes were extracted and plotted against the applied light intensity (Figure 7).

Higher light intensities led to stronger heating artifacts. The increase in artifact amplitude was fitted with a linear regression that was forced to start at the origin (0, 0), as no signal changes were expected without light application. At the lowest applied light intensity (19.5 mW/mm^2^) an average signal change of −0.4% was observed. Since optogenetic stimulation in this study was performed with 14.6–19.5 mW/mm^2^, we conclude that light application may diminish the BOLD response up to 0.4% relative to baseline signal. Our analysis in the small area around the fiber tip yields a realistic worst-case scenario. Importantly, the specific analysis may affect the estimation of light-induced signal changes especially at low light intensities. This is further detailed in Supplementary Figure 6.

### Signal Changes in Diffusion fMRI Are Similar to Those in GE-/SE-BOLD fMRI

**FIGURE 8:**
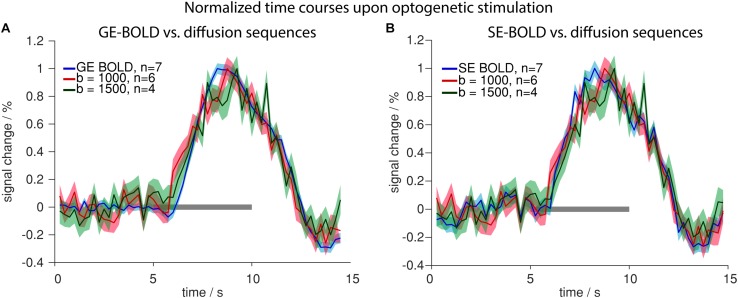
Comparison of BOLD and diffusion measurements. Normalized time courses upon optogenetic stimulation comparing GE-BOLD (blue) with diffusion with *b* = 1000 s/mm^2^ (red) and *b* = 1500 s/mm^2^ (green) **(A)** or SE-BOLD (blue) with diffusion with *b* = 1000 s/mm^2^ (red) and *b* = 1500 s/mm^2^ (green) **(B)** are shown. All time courses were acquired with TR 250 ms. Gray bars indicate stimulation periods, mean ± SEM is shown, n indicates number of measurements. All mean time courses are based on time courses extracted with the HRF GLM analysis.

Having characterized signal changes due to tissue heating, we next investigated the functional diffusion signal upon optogenetic stimulation. Signal changes were similar in GE-/SE-BOLD and diffusion measurements (Figure 8). Generally, the signal change started delayed after stimulation onset and reached its peak prior to end of stimulation. In contrast to GE-BOLD, signal from diffusion-weighted MRI did not show a signal undershoot after the end of the stimulation. There were no significant differences in time to peak for BOLD and diffusion measurements with *b* = 1000 s/mm^2^ (*p* > 0.1, *t*-test) or *b* = 1500 s/mm^2^ (no significance testing). The onset was slightly earlier for diffusion measurements with *b* = 1000 s/mm^2^ and *b* = 1500 s/mm^2^ compared to GE-BOLD. However, no significance testing was performed as less than four onset values could be calculated for the diffusion measurements. Onsets were also assessed for time courses obtained with the *U*-test analysis with 2 s or 0 s shift. Nearly identical time courses were obtained with the *U*-test analysis (Supplementary Figure 7). Significant onset differences were found for GE versus diffusion with *b* = 1000 s/mm^2^ (2 s shift) and GE and diffusion with *b* = 500 s/mm^2^ (0 s shift). No significant differences were observed between SE and diffusion sequences.

Summarizing these results, we conclude that voxelwise amplitudes were larger in diffusion than in BOLD measurements, while also SEM was larger for diffusion measurements due to the lower tSNR. No systematic differences in onset, time to peak or decay behavior were observed between diffusion and BOLD sequences.

### Neuronal Activation and Tissue Heating Lead to ADC Changes With Opposite Sign

**FIGURE 9:**
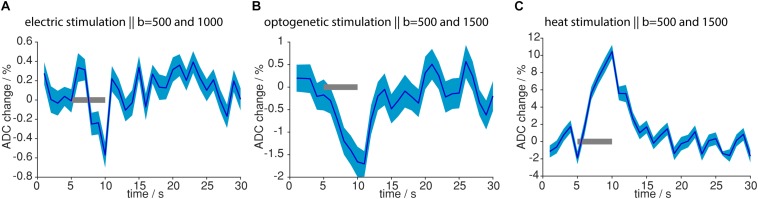
ADC changes upon different stimulations. **(A)** ADC change upon electric stimulation was calculated from 12 measurements with *b* = 1000 s/mm^2^ and 15 measurements with *b* = 500 s/mm^2^. **(B)** ADC change upon optogenetic stimulation was calculated from 4 measurements with *b* = 1500 s/mm^2^ and 7 measurements from *b* = 500 s/mm^2^. **(C)** ADC change upon light application was calculated from 18 measurements with *b* = 1500 s/mm^2^ and 16 measurements with *b* = 500 s/mm^2^. ADC change was most pronounced for light application. Gray bars indicate stimulation periods, mean and propagated error from SEM of averaged time courses are shown. ADCs were calculated from time courses based on the GLM analysis.

So far, MR signal from diffusion-weighted sequences was analyzed, which also carried T_2_- and T_2_^∗^-weighting, due to the EPI readout. To assess the isolated contribution of the diffusion component in the MR signal, we calculated the ADC. Upon both electric forepaw and optogenetic stimulation ADC decreased in response to the stimulation (Figure 9). On the contrary, during light application a strong (10.4%) increase in ADC was observed. These data clearly show that tissue heating can be ruled out as source of the functional diffusion signal.

## Discussion

In this study, we have investigated the response in both BOLD and diffusion fMRI experiments using fiber-based optical stimulation of the brain. Both types of readout differentiate between physiological signal changes and heating artifacts. We have shown that the observed heating artifacts are mostly due to T_2_^∗^-effects and that heating effects below the detection threshold may reduce the observed physiological signal changes of the BOLD response. We further characterized signal changes in functional diffusion measurements, and showed that these are not due to temperature effects.

### Heating Artifacts Have a Strong T_2_^∗^ Contribution

Heating artifacts can be a confounder in optogenetic fMRI studies. Their occurrence, extent and amplitude depend mainly on the mean light intensity applied to the tissue. However, the mechanisms behind these artifacts are still unclear. In agreement with previous studies (Christie et al., 2013; Schmid et al., 2017) we observed both positive and negative signal changes upon light application. Negative changes prevailed in terms of amplitude and extent. The appearance of such signal changes is due to competing effects of temperature on MR parameters (Rieke and Butts Pauly, 2008). Increased temperature leads to longer T_2_ relaxation times and, at 9.4 T, to shorter T_1_ relaxation times (de Graaf et al., 2006), both contributing to positive signal changes. However, light application may give rise to intra-voxel temperature gradients resulting in a decreased T_2_^∗^. Together with higher diffusion and smaller longitudinal magnetization at higher temperature, these changes contribute to negative signal changes.

In our measurements, GE-BOLD sequences showed larger areas of signal change compared to diffusion or SE-BOLD sequences. This observation suggests that rather T_2_^∗^ and not diffusion changes are the most prominent contributors to the observed signal changes. The spatial distribution of positive and negative signal changes in GE-BOLD measurements supports the notion of T_2_^∗^ relaxation as major mechanism. While negative signal changes dominated directly around the fiber, in neighboring slices positive and negative signal changes occurred in a mixed pattern.

Arias-Gil et al. have investigated tissue heating upon light application for optogenetic stimulation and found that the vascularity of the tissue had large impact on temperature. Twofold higher increases in temperature were observed when illuminating a blood vessel, as compared to illuminating tissue remote from visible blood vessels. This effect was explained by the high energy absorption of blood, which cannot be compensated by its heat diffusing capacity (Arias-Gil et al., 2016). The absorption coefficient of rat blood at 532 nm has been measured as 22.5 mm^–1^, which is about 74 time higher than the absorption coefficient of rat brain cortical tissue (0.3 mm^–1^) (Azimipour et al., 2015). Such large absorption differences between tissue and blood are likely to cause large temperature differences within individual voxels, resulting in intra-voxel dephasing and reduced T_2_^∗^ in the directly illuminated area around the fiber tip.

Distant from the fiber tip heating is expected to be weaker, because it most likely originates from heat conduction resulting in more uniform temperature profiles. While convection does not play a major role in heat distribution in tissue, conduction through micro-diffusion does (Stujenske et al., 2015). Stujenske et al. showed that models without heat diffusion severely overestimated the temperature increases due to light application via an optic fiber even for short illumination times. In that study, the temperature profile around the fiber was more uniform than the light penetration profile, which can be strongly influenced by absorbing vessels (Azimipour et al., 2015; Stujenske et al., 2015). Accordingly, the temperature effects further away from the fiber may be less governed by intra-voxel temperature gradients due to energy absorption, but better be explained by the expected bulk tissue changes in T_2_, T_1_ and magnetization with increasing temperature.

Besides tissue-specific MR parameters, physiological parameters like increased blood flow may be sources of heating artifacts. One study showed that light application with a mean intensity of 18 mW/mm^2^ led to hyperemia in the naïve rodent brain. The increase in blood flow was observed in ketamine-xylazine anesthetized mice and attributed to a decrease of calcium in the smooth muscle cells (Rungta et al., 2017). Recently, even an effect of light on neuronal cells has been shown. Owen et al. observed a decrease in firing activity in medium spiny neurons in striatum and in inhibitory neurons in cortex for constant illumination with 495 mW/mm^2^, the decrease in activity in medium spiny neurons was also significant at 99 mW/mm^2^ illumination (Owen et al., 2019). Accordingly, physiological effects of medium- and high-intensity light application to the brain are established on a cellular level. However, our opto-fMRI setup is most likely not sensitive to these, comparatively small, effects. We observed fundamentally different signal shapes between light application and sensory/optogenetic stimulation *in vivo*. In our *ex vivo* experiments we reproduced the signal shape upon high-intensity light application. Therefore, a relevant blood flow component can be excluded as source for heating artifacts. When performing electrophysiological experiments or calcium imaging experiments, the physiological effects of light may be relevant. Therefore, control experiments need to be performed for each specific setup.

The T_2_^∗^-effects described above may also apply *ex vivo*. Inhomogeneous microstructure of dead tissue may also cause intra-voxel dephasing close to the fiber tip, despite the absence of red blood cells. This notion is in agreement with the observation that higher light intensities were required to evoke heating artifacts in dead animals.

### Light Application Can Diminish BOLD Responses Upon Optogenetic Stimulation

In order to separate physiological responses from heating artifacts, we have assessed intensity limits for occurrence of heating artifacts. However, our analysis employed significance testing and the applied *p*-value threshold classified weak heating-induced signal changes as not significant. The effects of these subthreshold heating effects were revealed by a second analysis using a ROI directly around the fiber without significance testing. From these data, we estimated that subthreshold negative signal changes slightly diminish the physiological BOLD response upon optogenetic stimulation by up to 0.4%.

We used a light intensity of 162–217 mW/mm^2^ at the fiber tip and a duty cycle of 9% resulting in a mean light intensity of 14.6–19.5 mW/mm^2^. Other opto-fMRI studies have used lower light intensities. Takata et al. performed illumination of the skull with 1.1–2.5 mW and estimated that this led to an illumination as low as 0.002 mW/mm^2^ beneath the skull (Takata et al., 2018). Commonly an illumination of at least 1 mW/mm^2^ is used to reach sufficient illumination (Aravanis et al., 2007). Depending on the desired illumination volume, this will require higher light intensities at the fiber tip. For a light intensity of 1 mW/mm^2^ the estimated artifact in the small ROI around the fiber is negligible (−0.013%).

Besides temperature increases due to light application, inflow of warmer blood or radio frequency energy deposition by the MR sequence may affect brain temperature locally. In rodents, brain temperature may be lower than body temperature and inflow of warmer blood during sensory stimulation may reduce the BOLD response by up to 1%, as previously estimated from phantom experiments (Harris et al., 2018). In the case of optogenetic fMRI, brain-body temperature gradients may be kept low by avoiding large craniotomies or cranial windows. Further, by using opsins with high light-sensitivity or red-shifted activation wavelengths, heating from light application can be minimized (Kleinlogel et al., 2011; Yizhar et al., 2011; Klapoetke et al., 2014; Ganjawala et al., 2019).

### Signal Changes in Diffusion-Type Measurements Are Not a Heating Effect but Depend on Changes in Diffusion, T_2_ and T_2_^∗^

Our characterization of signal changes in functional diffusion measurements resulted in two major observations. First, signal time courses acquired using a single SE diffusion-weighted sequence, closely followed the response in GE-BOLD measurements. Second, ADC changes upon sensory stimulation were opposite to those during tissue heating.

We acquired time courses with 250 ms temporal resolution to compare temporal evolutions of signal changes in diffusion and BOLD fMRI. Time courses of diffusion fMRI showed a hemodynamic response-like behavior, following the signal evolution of GE- and SE-BOLD fMRI. Both diffusion and BOLD fMRI signals started delayed and decreased slowly after the peak. We did not detect time to peak differences between BOLD and diffusion fMRI. This is in contrast to one study in rats in which the diffusion fMRI signal, measured at a TR of 1.5 s, peaked earlier than the BOLD fMRI signal (1.8 ± 0.6 s versus 3.3 ± 0.6 s) (Tsurugizawa et al., 2013). Another recent study using a line scanning scheme with 100 ms temporal resolution did not detect an earlier time to peak but found an early signal change component in functional diffusion (Nunes and Shemesh, 2019). This component appeared less than 200 ms after stimulation onset. We assume that the temporal resolution in the present study was not sufficient to resolve these fast components of the functional diffusion signal.

The hemodynamic response-like behavior of the diffusion signal observed in our study is most likely due to the T_2_- and T_2_^∗^-weighting of the employed single SE diffusion-weighted EPI sequence. T_2_^∗^-weighting is intrinsic to the EPI sequence, resulting in an important confounder in diffusion-weighted fMRI. While conventional SE sequences are T_2_-weighted, SE-EPI sequences are both T_2_- and T_2_^∗^-weighted due to the EPI readout, which adds T_2_^∗^-weighting. The strength of T_2_^∗^-weighting is expected to be substantial, since the employed EPI readout time of 25.6 ms was in the same range as the T_2_^∗^ of rat brain gray matter at 9.4 T. One previous study found that the EPI readout time needed to be reduced to half of the T_2_ time of the brain, to become more sensitive to micro- than macro-vasculature (Goense and Logothetis, 2006).

To mitigate the T_2_/T_2_^∗^-weighting the diffusion component of the signal can be isolated by calculating the ADC. We found negative ADC changes upon sensory and optogenetic stimulation but positive ADC changes during high-intensity light application. Accordingly, the ADC changes upon sensory/optogenetic stimulation are not caused by tissue heating. High quality ADC data requires diffusion sequences that are robust against background B_0_ gradients. These background gradients may arise due to susceptibility differences caused by inhomogeneities within the tissue or by changes in the oxy/deoxyhemoglobin ratio upon activation. Background gradients add to the diffusion gradients and hinder a precise definition of the actual *b*-value. The single SE preparation is prone to interactions of the diffusion gradients with background gradients. We used very short strong diffusion gradients and accordingly very short echo times for single SE preparation to minimize the interaction time with background gradients (Pampel et al., 2010). However, double SE sequences or oscillating gradients with odd gradient symmetry may suppress the effect of background gradients even better (Schachter et al., 2000). Especially at lower field strengths double SE preparations should be used to counteract the effects of background gradients. Longer TE, however, decreases SNR and thus compromises quality of ADC data. Double SE preparation therefore requires specialized hardware offering higher field strength, better gradient performance or cryogenic coils (Nunes et al., 2019). Such advances may be employed to further elucidate the origin of the diffusion fMRI signal or to characterize response to optogenetic stimulation in more detail.

## Conclusion

In this study we investigated the functional MRI signal in GE-BOLD, SE-BOLD and diffusion fMRI measurements and characterized heating artifacts, which may be confounders in optogenetic fMRI studies. Analysis of data obtained with the different MRI sequences showed that heating artifacts are mainly based on T_2_^∗^-effects and show a specific signal shape that differs from the hemodynamic response-like signal shape upon sensory and optogenetic stimulation. Further analysis revealed that the BOLD response in GE-BOLD during light application may be diminished, already at the low light intensities used for optogenetic stimulation.

In diffusion fMRI measurements, the observed signal followed the signal shape of BOLD fMRI, since single SE diffusion sequences were dominated by relaxation time rather than diffusion effects. Accordingly, ADC calculation was required to observe diffusion changes upon sensory and optogenetic stimulation. Heating effects were excluded as a contributor to the observed changes in functional ADC measurements. Detailed characterization of the time course of ADC changes to provide novel insight into the mechanisms of the diffusion fMRI signal or to further characterize the response to optogenetic stimulation will require dedicated hardware.

## Data Availability Statement

The raw data supporting the conclusions of this manuscript will be made available by the authors, without undue reservation, to any qualified researcher.

## ETHICS STATEMENT

The animal study was reviewed and approved by Landesamt für Natur, Umwelt und Verbraucherschutz of Nordrhein-Westfalen, Germany.

## Author Contributions

FA and CF designed the study. FA, DS, HL, and LW conducted the fMRI experiments. FA analyzed the data. HL established the *U*-test analysis. FA, LW, and CF wrote the manuscript. All authors edited and approved the manuscript.

## Conflict of Interest

The authors declare that the research was conducted in the absence of any commercial or financial relationships that could be construed as a potential conflict of interest.
