# Supplementary material for: Functional MRI Readouts From BOLD and Diffusion Measurements Differentially Respond to Optogenetic Activation and Tissue Heating
Source: Front Neurosci. 2019 Oct 24;13:1104. doi: 10.3389/fnins.2019.01104 (PMC6821691; doi:10.3389/fnins.2019.01104)
Supplement: Supplementary file 1 [file Data_Sheet_1.docx]

Supplementary Material for:

Functional MRI readouts from BOLD and diffusion measurements differentially respond to optogenetic activation and tissue heating

Franziska Albers^1^, Lydia Wachsmuth^1^, Daniel Schache^1^, Henriette Lambers^1^, Cornelius Faber^1^

^1^ Translational Research Imaging Center (TRIC), Department of Clinical Radiology, University Hospital Münster, Germany

# Supplementary Figures

## Data analysis using the U-tests: statistical testing to determine activated voxels


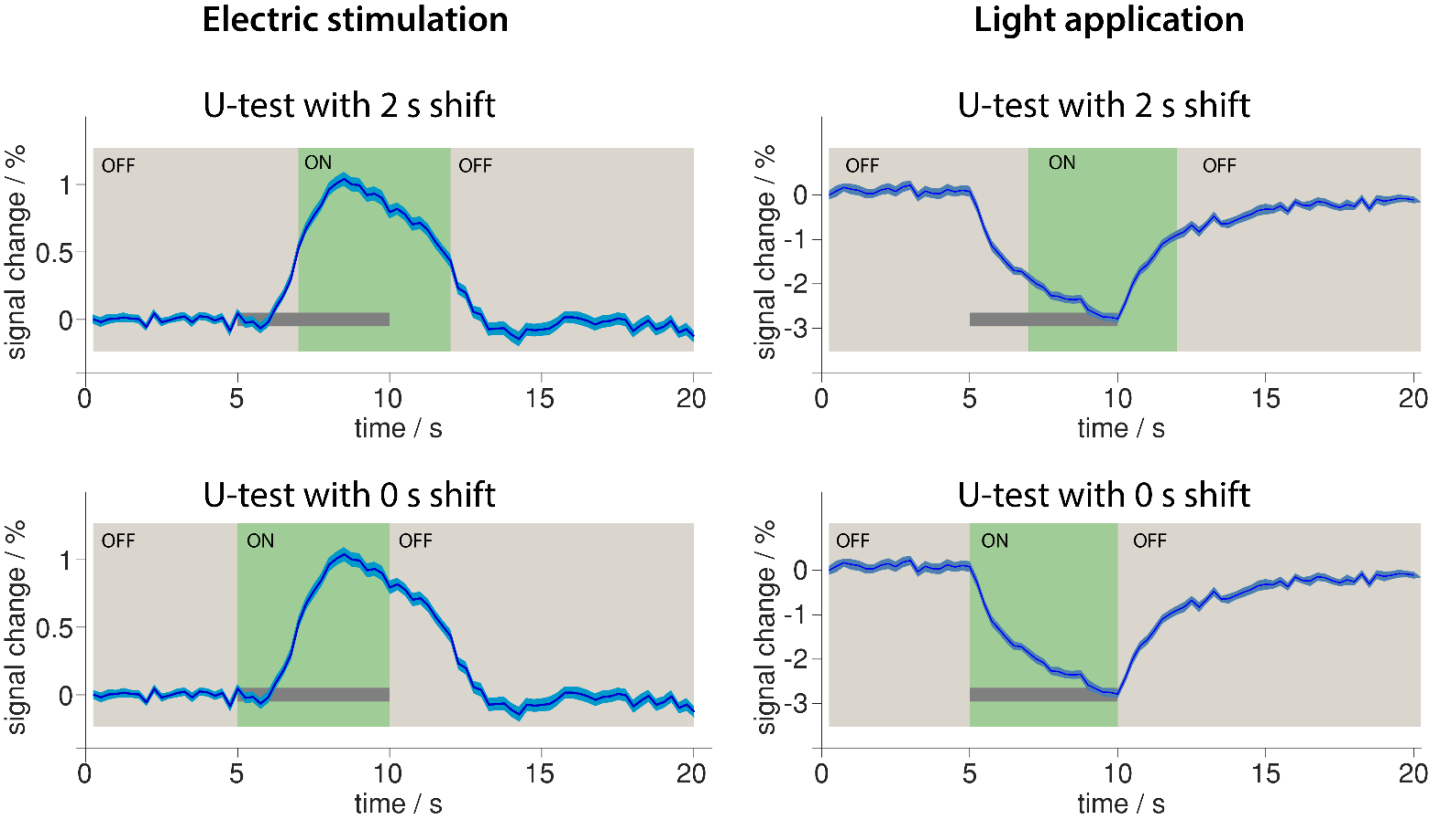


**Supplementary Figure 1: Schematics of stimulation periods used for the U-test to determine activated voxels.** Exemplary time courses from GE-BOLD sequences for electric stimulation (left) and high-intensity light application (right) are shown. In the standard analysis the U-test was performed using a stimulation period (green box) shifted by 2 s (upper panels). The alternative analysis used a stimulation period that was not shifted (lower panels). Grey bars at the bottom indicate actual stimulation periods, green and grey boxes indicate stimulation and rest times as used for the U-test, respectively.

## Comparison of time courses based on GLM and U-test analysis (2 s shift)


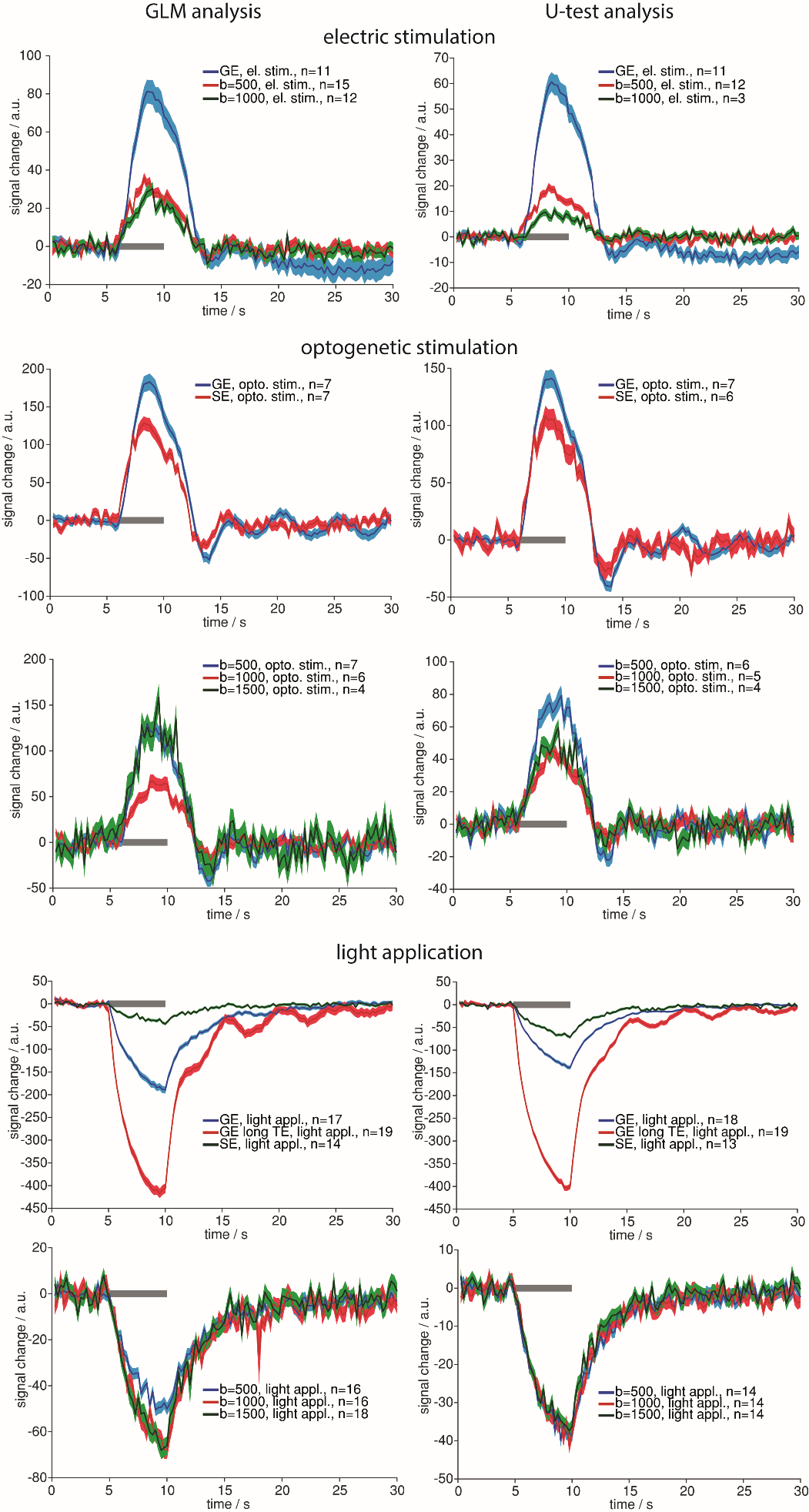


**Supplementary Figure 2:** All in vivo area-weighted time courses based on GLM analysis (left) and U-test analysis with 2 s shift (right). Grey bars indicate stimulation periods, mean ± SEM is shown, n indicates number of measurements.

## Extent of heating artifacts is larger in GE-BOLD than in SE or diffusion sequences

Maps of significantly altered signal in the brain upon either optogenetic stimulation or high-intensity light application were calculated using the U-test procedure, as described in the main text. These maps showed distinctly larger activation clusters for GE-BOLD than for diffusion sequences upon high-intensity light application (Supplementary Figure 3 and 4).

To take both cluster size and voxel-wise amplitude into account, area-weighted time courses were calculated by multiplying the individual time courses from each animal with the number of activated voxels before averaging. The area-weighted signal change was largest for GE-BOLD (Supplementary Figure 4a) and lowest for diffusion with b=1000 s/mm² (Supplementary Figure 4c).


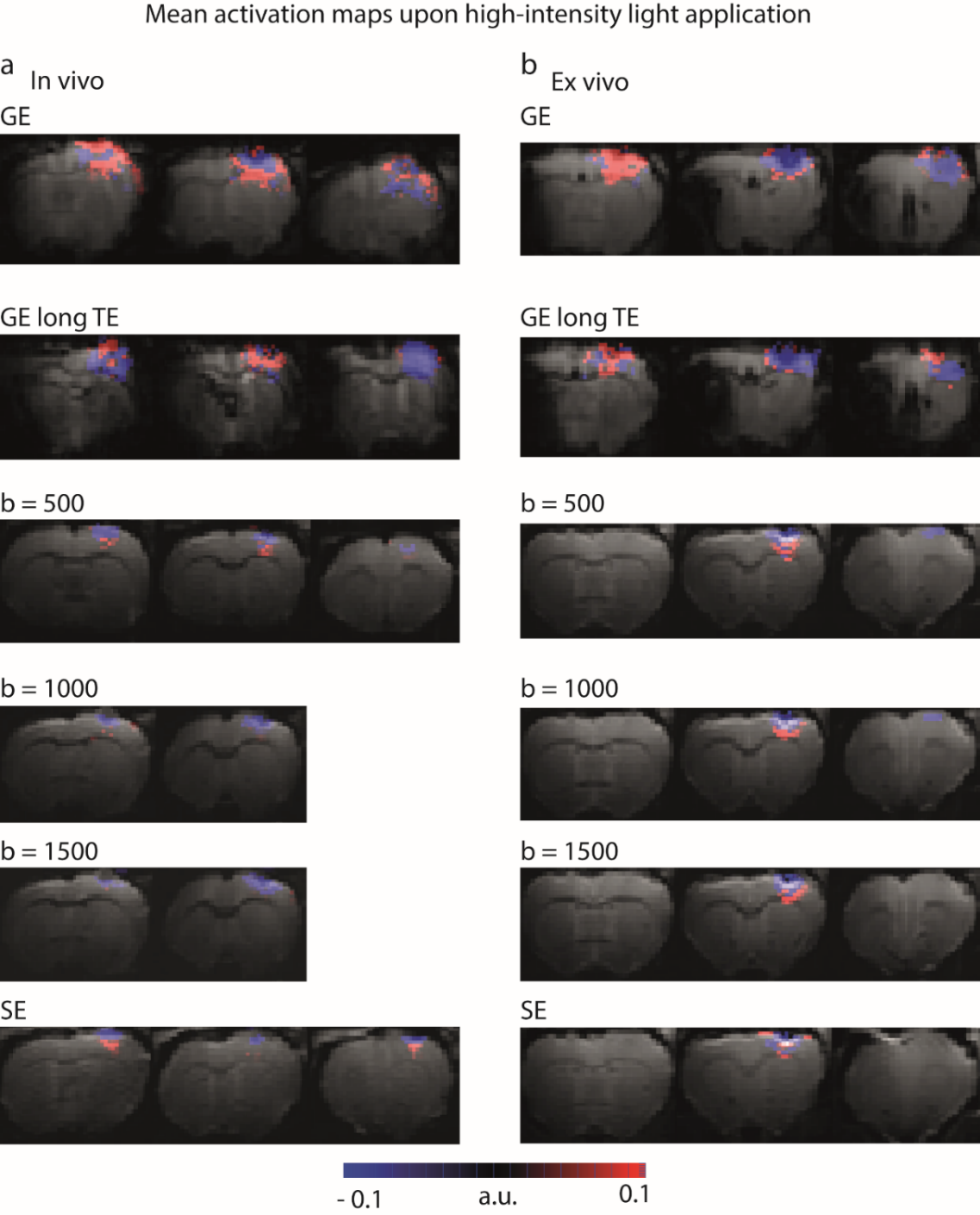


**Supplementary Figure 3**: Mean activation maps upon high-intensity light application in vivo (a) and ex vivo (b). Mean activation maps were calculated from maps generated with the U-test analysis with 2 s shift. The color bar applies to all maps.


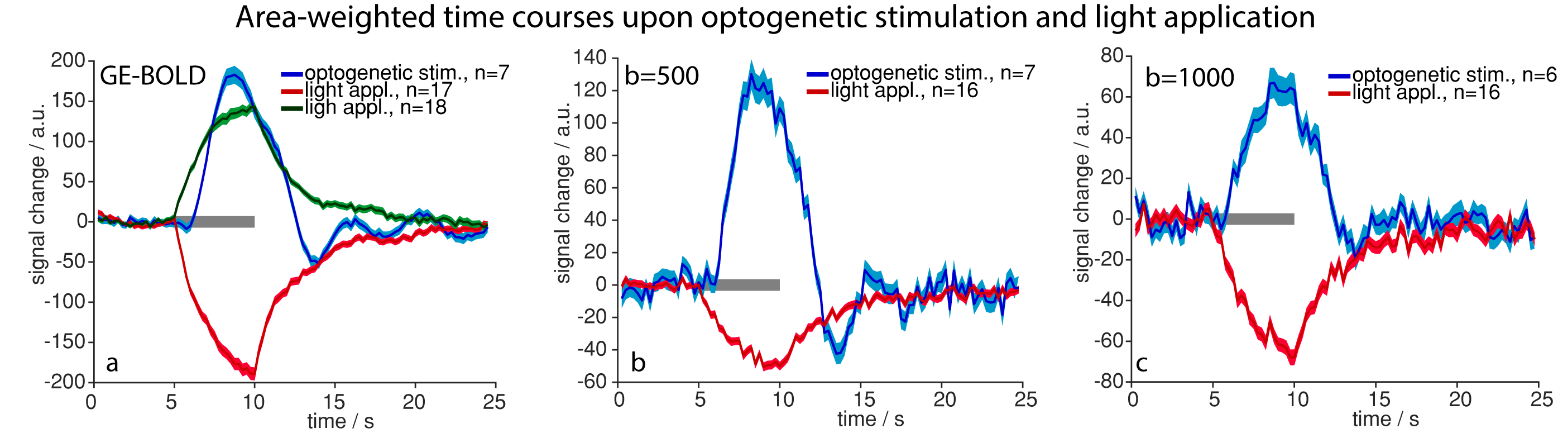


**Supplementary Figure 4:** Area-weighted time courses for optogenetic stimulation and high-intensity light application for GE-BOLD (a), diffusion with b=500 s/mm² (b) and diffusion with b=1000 s/mm² (c) are shown. Data were analyzed with the GLM approach. Grey bars indicate stimulation periods, mean ± SEM is shown, n indicates number of measurements. Note different scales in A, B and C; all plots use identical units.

## Mean activation maps for electric and optogenetic stimulation

Mean activation maps based on the single maps of the U-test analysis with 2 s shift were calculated. For all types of stimulation, GE sequences reveal largest areas of activation.


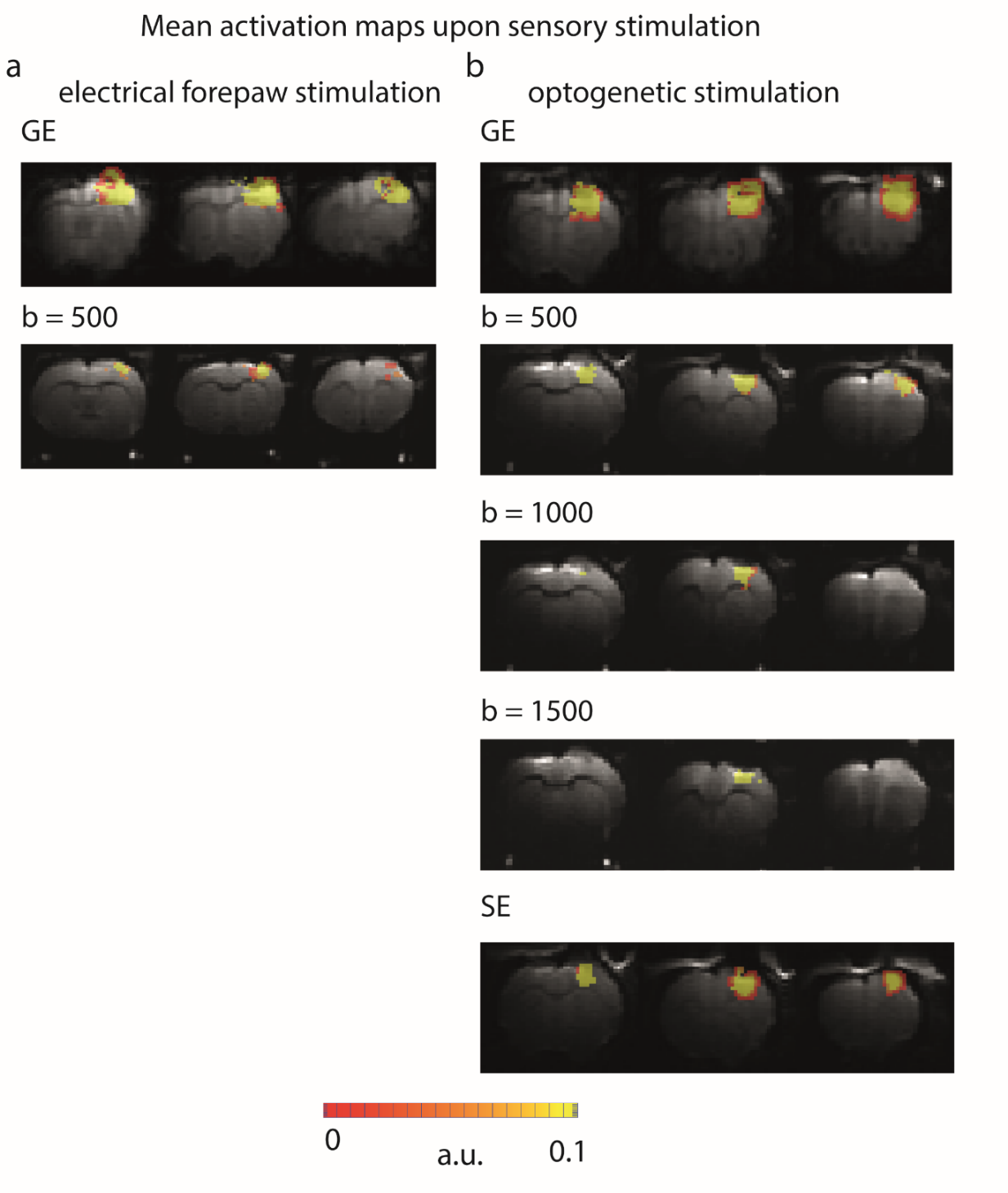


**Supplementary Figure 5**: Mean activation maps upon electric (a) and optogenetic stimulation (b). Mean activation maps were calculated from maps generated with the U-test analysis with 2 s shift. The color bar applies to all maps.

## Threshold for heating artifacts is experiment- and analysis-specific

As described in the main text, light applications with mean intensities between 19.5 mW/mm² and 195 mW/mm² were performed in five naïve animals. In the main text these data were used for two analyses. First, the analysis was performed as described in “U-test analysis” using a voxelwise U-test in a ROI determined by a preceding not corrected t-test. If at least five significantly activated voxels were found, data were averaged across voxels and stimulation trials. Negative signal changes were extracted and plotted against the applied light intensity. No heating artifacts were found for a light intensity of 19.5 mW/mm² (as shown in Table1 in the main text) which was then defined as a safe limit for the optogenetic stimulation.

Second, these data were analyzed without using significance testing as described in “Additional Analysis Without Significance Testing for Experiment 1” in the main text. A small ROI directly around the fiber was chosen, data were averaged across the ROI and across stimulation trials. This analysis revealed negative activation (0.4 %) in the area directly around the fiber at 19.5 mW/mm² and led us to conclude that subthreshold heating artifacts are present for low light intensities (Figure 7 in the main text). The difference between both methods is explained by the significance testing used in the first analysis procedure, which introduced a cut-off value in the analyzed data (Supplementary Figure 6A). When a statistical threshold was defined, weak heating artifacts were classified as not significant. Yet, these signal changes were present in the measured data. These subthreshold artifacts were preserved by the second analysis procedure, using a small ROI around the fiber without significance testing.

To further characterize the effect of the analysis we modified the fixed-ROI analysis. A larger ROI defined anatomically to include the somatosensory cortex in the slice with the fiber was chosen. Data were averaged across the ROI and stimulation trails, without performing any statistical testing (Supplementary Figure 6B). A linear regression was forced through the origin, since no signal changes were postulated without application of light. The resulting effect on signal changes for light intensities as used for optogenetic stimulation was much smaller than with the small ROI, since signal was averaged across a large area with a number of voxels not containing any signal changes.

We therefore conclude that the choice of analysis affects the measured threshold for heating artifacts. Accordingly, future control experiments in optogenetic studies should be devised individually, taking the experimental setup including the analysis pipeline into account.


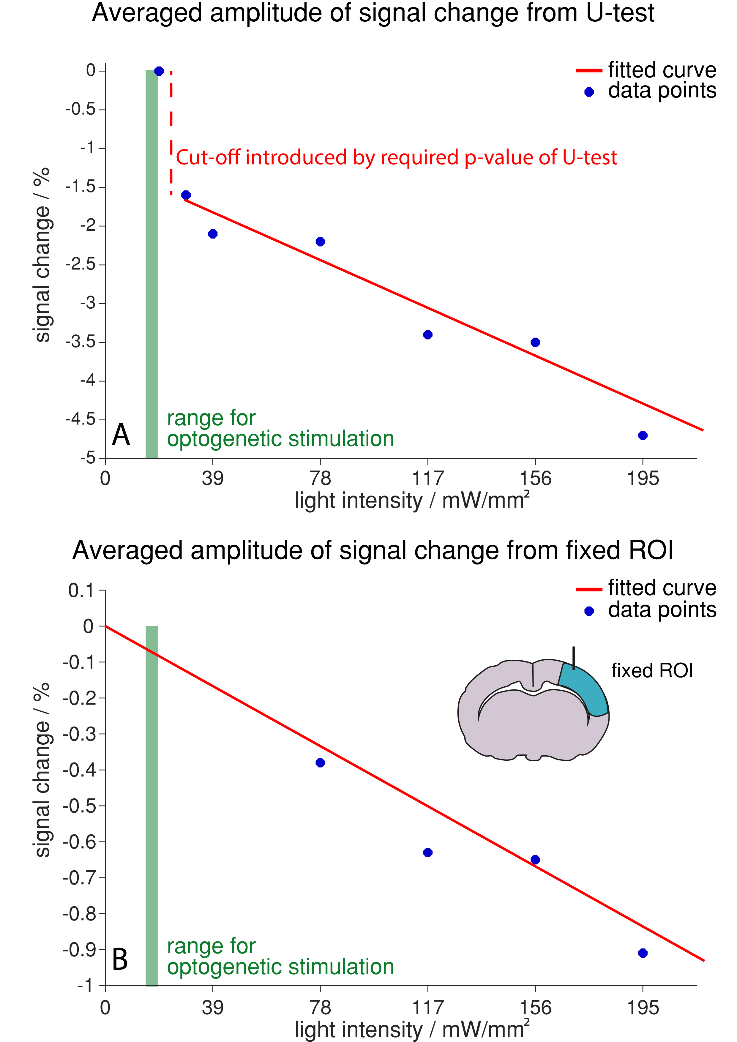


**Supplementary Figure 6** Peak amplitudes of negative signal change upon light application plotted against applied light intensity. Solid red line indicates a linear regression performed on the data. Green box indicates range of light intensities for optogenetic stimulation in this study. In (A) data analyzed with a U-test are shown. The calculated fit was f(x)=-0.017x -1.17. In (B) data averaged across a fixed ROI without any significance testing are shown. For this data, the fit was forced to include the origin (0, 0) and resulted in f(x)=-0.005x.

## Comparison of diffusion and BOLD fMRI signal shape based on the U-test analysis


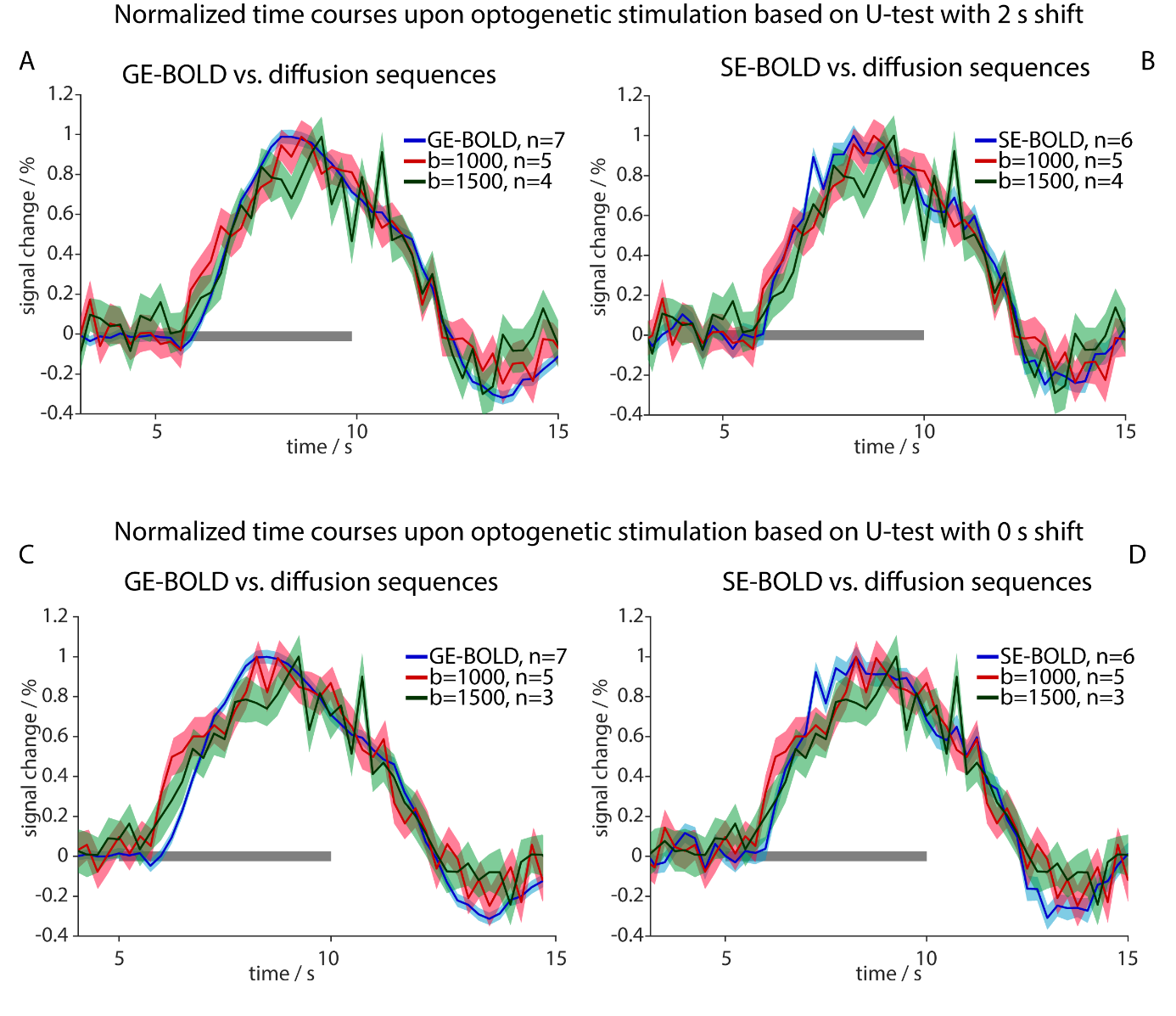


**Supplementary Figure 7:** Normalized time courses upon optogenetic stimulation. In (A,B) time courses from U-test analysis with 2 s shift and in (C,D) time courses from analysis with 0 s shift are shown. (A,C) GE-BOLD (blue) compared with diffusion with b=1000 s/mm² (red) and b=1500 s/mm² (green). (B,D) SE-BOLD (blue) compared with diffusion with b=1000 s/mm² (red) and b=1500 s/mm² (green). All time courses were acquired with TR 250 ms. Grey bars indicate stimulation periods, mean ± SEM is shown, n indicates number of measurements.

# Supplementary Tables

**Supplementary Table 1:** Overview over averaged amplitudes and area-weighted amplitudes of time courses measured in vivo from GLM based and U-test based analysis. Averages are shown when at least five measurements were available. SEM is given as error. Mean activated voxel is the mean of measurements with more than 5 activated voxels.

|  | GLM-based | | | U-test-based (2 s shift) | | | |
| --- | --- | --- | --- | --- | --- | --- | --- |
|  | Averaged amplitude | Area-weighted amplitude | Mean activated voxel | Averaged amplitude | Area-weighted amplitude | Mean activated voxel |  |
|  | % | a.u. |  | % | a.u. |  |  |
| GE el | 0,86 ± 0,04 | 77,97 ± 4,36 | 87,60 | 1,11 ± 0,04 | 63,92 ± 0,06 | 54,30 |  |
| b = 500 el | 1,77 ± 0,12 | 35,90 ± 2,60 | 22,90 | 2,50 ± 0,17 | 19,60 ± 1,50 | 9,30 |  |
| b = 1000 el | 1,90 ± 0,18 | 30,40 ± 3,30 | 16,20 |  |  |  |  |
| GE opto | 1,31 ± 0,05 | 183,00 ± 11,00 | 125,30 | 1,67 ± 0,06 | 141,28 ± 8,12 | 76,70 |  |
| SE opto | 2,64 ± 0,15 | 128,50 ± 9,10 | 51,50 | 3,56 ± 0,18 | 108,36 ± 8,83 | 41,00 |  |
| b = 500 opto | 2,36 ± 0,15 | 130,02 ± 9,16 | 51,10 | 3,41 ± 0,22 | 79,20 ± 6,33 | 31,40 |  |
| b = 1000 opto | 3,07 ± 0,25 | 66,86 ± 7,46 | 26,30 | 4,95 ± 0,41 | 45,43 ± 0,41 | 12,75 |  |
| GE light (neg) | -1,56± 0,04 | -189,19 ± 8,07 | 124,06 | -2,85 ± 0,06 | -138,62 ± 4,14 | 52,94 |  |
| GE long TE light (neg) | -3,60 ± 0,07 | -417,15 ± 10,47 | 118,68 | -6,89 ± 0,10 | -403,76 ± 6,01 | 59,47 |  |
| SE light (neg) | -3,27 ± 0,21 | -44,01 ± 3,08 | 15,14 | -6,82 ± 0,28 | -71,66 ± 3,00 | 11,00 |  |
| b = 500 light (neg) | -2,22 ± 0,11 | -50,55 ± 2,70 | 23,00 | -4,76 ± 0,18 | -39,90 ± 1,63 | 9,14 |  |
| b = 1000 light (neg) | -2,86 ± 0,16 | -67,92 ± 0,16 | 23,38 | -5,49 ± 0,25 | -41,33 ± 1,89 | 7,93 |  |
| b = 1500 light (neg) | -3,45 ± 0,19 | -68,06 ± 3,83 | 19,83 | -6,93 ± 0,35 | -37,41 ± 1,94 | 6,91 |  |
| GE light (pos) | 1,00 ± 0,03 | 142,85 ± 5,72 | 153,22 | 1,46 ± 0,04 | 84,33 ± 2,90 | 56,22 |  |
| GE long TE light (pos) | 1,92 ± 0,06 | 172,86 ± 7,39 | 85,84 | 4,22 ± 0,11 | 175,30 ± 7,08 | 37,58 |  |
| SE light (pos) | 1,95 ± 0,21 | 17,61 ± 2,01 | 10,44 | 3,93 ± 0,39 | 25,19 ± 2,44 | 8,50 |  |
| b = 500 light (pos) | 1,49 ± 0,16 | 18,07 ± 1,95 | 13,50 |  |  | 8,00 |  |
| b = 1000 light (pos) |  |  | 7,00 |  |  | 6,00 |  |
| b = 1500 light (pos) |  |  | 14,00 |  |  | 0,00 |  |

**Supplementary Table 2:** Time to peak and time to baseline values for mean time courses based on U-test with 2 s shift analysis. Time to baseline refers to the time from the start of the stimulation until the baseline is reached again after the peak. Error of time to peak and time to baseline was estimated by the relative error determined by the SEM of the peak amplitude. Times to peak and times to baseline were shorter for responses upon electric/optogenetic stimulation compared to responses upon light application.

|  | Time to peak | Time to baseline |
| --- | --- | --- |
|  | s | s |
| GE el | 3,50 ± 0,12 | 12,50 ± 0,45 |
| B=500 el | 3,25 ± 0,22 | 13,00 ± 0,88 |
| GE opto | 3,25 ± 0,11 | 12,50 ± 0,42 |
| SE opto | 3,25 ± 0,17 | 12,50 ± 0,65 |
| B=500 opto | 3,25 ± 0,21 | 12,50 ± 0,81 |
| B=1000 opto | 3,75 ± 0,31 | 12,25 ± 1,01 |
| GE light | 5,00 ± 0,11 | 25,75 ± 0,56 |
| GE long TE light | 4,75 ± 0,07 | 25,75 ± 0,38 |
| SE light | 5,00 ± 0,20 | 21,25 ± 0,87 |
| B=500 light | 4,00 ± 0,15 | 25,25 0±,97 |
| B=1000 light | 4,75 ± 0,21 | 19,25 ± 0,86 |
| B=1500 light | 4,75 ± 0,24 | 17,00 ± 0,86 |
